# Robust segmentation of lung in chest x-ray: applications in analysis of acute respiratory distress syndrome

**DOI:** 10.1186/s12880-020-00514-y

**Published:** 2020-10-15

**Authors:** Narathip Reamaroon, Michael W. Sjoding, Harm Derksen, Elyas Sabeti, Jonathan Gryak, Ryan P. Barbaro, Brian D. Athey, Kayvan Najarian

**Affiliations:** 1 Department of Computational Medicine and Bioinformatics, University of Michigan, Ann Arbor, MI USA; 2 Department of Internal Medicine, University of Michigan, Ann Arbor, MI USA; 3 Department of Mathematics, University of Michigan, Ann Arbor, MI USA; 4 Michigan Institute for Data Science, University of Michigan, Ann Arbor, MI USA; 5 Department of Pediatrics, University of Michigan, Ann Arbor, MI USA

**Keywords:** Lung segmentation, Chest x-ray, Acute respiratory distress syndrome

## Abstract

**Background:**

This study outlines an image processing algorithm for accurate and consistent lung segmentation in chest radiographs of critically ill adults and children typically obscured by medical equipment. In particular, this work focuses on applications in analysis of acute respiratory distress syndrome – a critical illness with a mortality rate of 40% that affects 200,000 patients in the United States and 3 million globally each year.

**Methods:**

Chest radiographs were obtained from critically ill adults (n = 100), adults diagnosed with acute respiratory distress syndrome (ARDS) (n = 25), and children (n = 100) hospitalized at Michigan Medicine. Physicians annotated the lung field of each radiograph to establish the ground truth. A Total Variation-based Active Contour (TVAC) lung segmentation algorithm was developed and compared to multiple state-of-the-art methods including a deep learning model (U-Net), a random walker algorithm, and an active spline model, using the Sørensen–Dice coefficient to measure segmentation accuracy.

**Results:**

The TVAC algorithm accurately segmented lung fields in all patients in the study. For the adult cohort, an averaged Dice coefficient of 0.86 ±0.04 (min: 0.76) was reported for TVAC, 0.89 ±0.12 (min: 0.01) for U-Net, 0.74 ±0.19 (min: 0.15) for the random walker algorithm, and 0.64 ±0.17 (min: 0.20) for the active spline model. For the pediatric cohort, a Dice coefficient of 0.85 ±0.04 (min: 0.75) was reported for TVAC, 0.87 ±0.09 (min: 0.56) for U-Net, 0.67 ±0.18 (min: 0.18) for the random walker algorithm, and 0.61 ±0.18 (min: 0.18) for the active spline model.

**Conclusion:**

The proposed algorithm demonstrates the most consistent performance of all segmentation methods tested. These results suggest that TVAC can accurately identify lung fields in chest radiographs in critically ill adults and children.

## Background

Lung segmentation, the process of accurately identifying regions and boundaries of the lung field from surrounding thoracic tissue, is an essential first step in pulmonary image analysis of many clinical decision support systems. Correct identification of lung fields enables further computational analysis of these anatomical regions [1], such as extraction of clinically relevant features to train a machine learning algorithm for detection of disease and abnormalities. These computational methodologies can assist physicians with making a timely, accurate medical diagnosis to improve quality of care and outcome for patients.

Although many methods exist in the literature [2–8], they are primarily designed and evaluated on high quality, standardized chest radiographs from controlled studies or outpatient settings that may not be representative of more complex chest x-rays (CXR) from hospitalized patients. This is problematic for many patient populations, especially the critically ill, whose CXRs tend to have characteristics of varying image quality (e.g. dynamic range, sharpness), presence of introduced medical devices [9], diverse body habitus [10], and manifestation of disease [11]. As a result, these methods may not generalize and perform as well on chest x-rays (CXR) obtained from other clinical settings.

We therefore hypothesize that it may be possible to use image processing techniques to handle heterogeneous characteristics of CXRs to facilitate better, more generalizable lung segmentation. The aim of this study was to develop such an algorithm capable of robust and reliable performance on multiple patient populations, including critically ill patients. Our proposed hierarchical method first uses total variation denoising to remove irrelevant details and artifacts from medical equipment obscuring the lung fields. The image is then binarized with recursive thresholding to identify the left and right lung fields. Finally, a stacked active contour model is used to refine the final shape of the segmentation mask. The proposed method also incorporates systematic quality checks by using various assessment criteria at each step to ensure consistent, successful segmentation. It is especially important that these clinical decision support systems are highly reliable to ensure healthcare providers that the algorithm will consistently perform as expected, even in the most rigorous tasks.

We evaluate this method on multiple datasets, including two publicly available CXR repositories and data from Michigan Medicine comprising of critically ill patients with respiratory failure. Furthermore, we compare the proposed algorithm’s performance to multiple state-of-the-art lung segmentation methods, including a deep learning approach [12], standard computer vision algorithms [13], and conventional image processing techniques [14].

## Methods

### Dataset and study population

The Institutional Review Board approved this study with a waiver of informed consent. We retrospectively identified three cohorts of patients hospitalized in adult and pediatric intensive care units at Michigan Medicine in 2016 and 2017. The first cohort was a random sample of 100 adult patients (mean age 58 years ±16 [standard deviation], 48% female) with acute hypoxic respiratory failure (PaO2/FiO2 ratio of <300 mm Hg while receiving invasive mechanical ventilation), stratified such that 50 of the patients met the criteria for the Acute Respiratory Distress Syndrome (ARDS) after review by clinical experts. The second cohort included chest radiographs from 25 additional adult patients (mean age 55 years ±17 [standard deviation], 44% female) with “high confidence ARDS” by multiple physicians [15]. Chest radiographs from this cohort would be expected to have intense, widespread bilateral opacities that would be more difficult for segmentation algorithms. The third cohort included 100 chest x-rays from pediatric patients (mean age 7 years ±5 [standard deviation], 39% female) hospitalized in the Pediatric Intensive Care Unit. Children age 14 days to 19 years with an endotracheal tube on mechanical ventilation were eligible for inclusion; this cohort was stratified such that 50 of the patients met criteria for pediatric ARDS. Additional details of these patient groups are provided in Table 1.

**Table 1:** Patient Demographic of Michigan Medicine Cohorts

	**n**	**Age**	**Non-ARDS**	**ARDS**
**Adult Cohort**				
**Total**	100	58 ±16	50	50
**Male**	52	60 ±16	30	22
**Female**	48	54 ±16	20	28
**Adult Severe ARDS Cohort**				
**Total**	25	55 ±17	0	25
**Male**	14	56 ±16	0	14
**Female**	11	53 ±19	0	11
**Pediatric Cohort**				
**Total**	100	7 ±5	50	50
**Male**	61	9 ±5	31	30
**Female**	39	6 ±6	19	20

A total of 225 anterior-posterior chest radiographs were exported from Michigan Medicine’s picture archiving and communication system then stored in the Digital Imaging and Communications in Medicine format prior to analysis. Annotations for ground truth of the lung regions on the two adult patient groups were performed by a pulmonary critical care physician with 4 years of clinical experience. Annotations for the pediatric cohort were performed by a pediatric critical care intensivist with 5 years of clinical experience.

Chest radiographs from two publicly available datasets were also used to validate the algorithm in other patient populations. The Japanese Society of Radiological Technology (JSRT) [16, 17] is comprised of 247 posterior-anterior chest x-rays: 154 containing a pulmonary lung nodule and the remaining 93 without any nodules. The second external dataset from Montgomery County, made available by the U.S. National Library of Medicine [18], contains 138 posterior-anterior chest x-rays: 58 are cases with manifestations of tuberculosis and the remaining 80 are representative of normal, healthy lungs. A summary of these patient groups is provided in Table 2.

**Table 2:** Patient Demographic of JSRT and Montgomery Datasets

	**n**	**Normal**	**Abnormal**
**JSRT**			
**Total**	247	93	154
**Male**	119	n/a	n/a
**Female**	128	n/a	n/a
**Montgomery**			
**Total**	138	80	58
**Male**	64	n/a	n/a
**Female**	74	n/a	n/a

Individual patient age and gender information were not available for these two publicly available databases. In the JSRT dataset, “abnormal” refers to the presence of lung nodules. In the Montgomery dataset, “abnormal” refers to the manifestation of tuberculosis.

### Proposed method: total variation-based active contour (TVAC)

The proposed algorithm, Total Variation-based Active Contour (TVAC), is comprised of three primary steps. Total variation denoising was employed to delineate and remove various medical equipment visually obscuring the lung fields. A recursive binarization method was used to systematically identify the lungs and a stacked active contour model was utilized to improve lung boundary formation. Prior to execution, chest radiographs are first normalized with contrast-limited adaptive histogram equalization (CLAHE) [19] to adjust contrast locally while limiting the amplification of noise to ensure that x-rays in the dataset are generally represented within the same pixel intensity range.

#### Total variation denoising

Total Variation Denoising is a method to remove noise from images using a model of Rudin, Osher and Fatemi (ROF) [20]. If $f:\Omega \to \mathbb {R}$ is a grayscale image, where *Ω* is a rectangle in $\mathbb {R}^{2}$, then the total variation of *f* is:

1 $$ \begin{aligned} \|f\|_{\text{TV}}=\int_{\Omega} |\nabla f| \end{aligned}  $$

To denoise an image *f*, we find an approximation *u* of *f* for which ∥*u*∥_TV_ is small by minimizing:

2 $$ \lambda\|u\|_{\text{TV}}+\frac{1}{2}\int_{\Omega}(f-u)^{2}  $$

Here *λ* is a regularization parameter. The level sets of an optimal solution *u* have a small perimeter (relative to their area) (see for example [21, §2.2.2]). This means that boundaries of the level sets tend to be smooth and round. ROF denoising removes local details in images, while maintaining and smoothing the boundaries of larger areas.

There are many algorithms for solving the optimization problem in the ROF model. We use an algorithm and implementation of Zhu and Chan [22] that uses the Primal-Dual Hybrid Gradient method (PDHG).

**Fig. 1:**
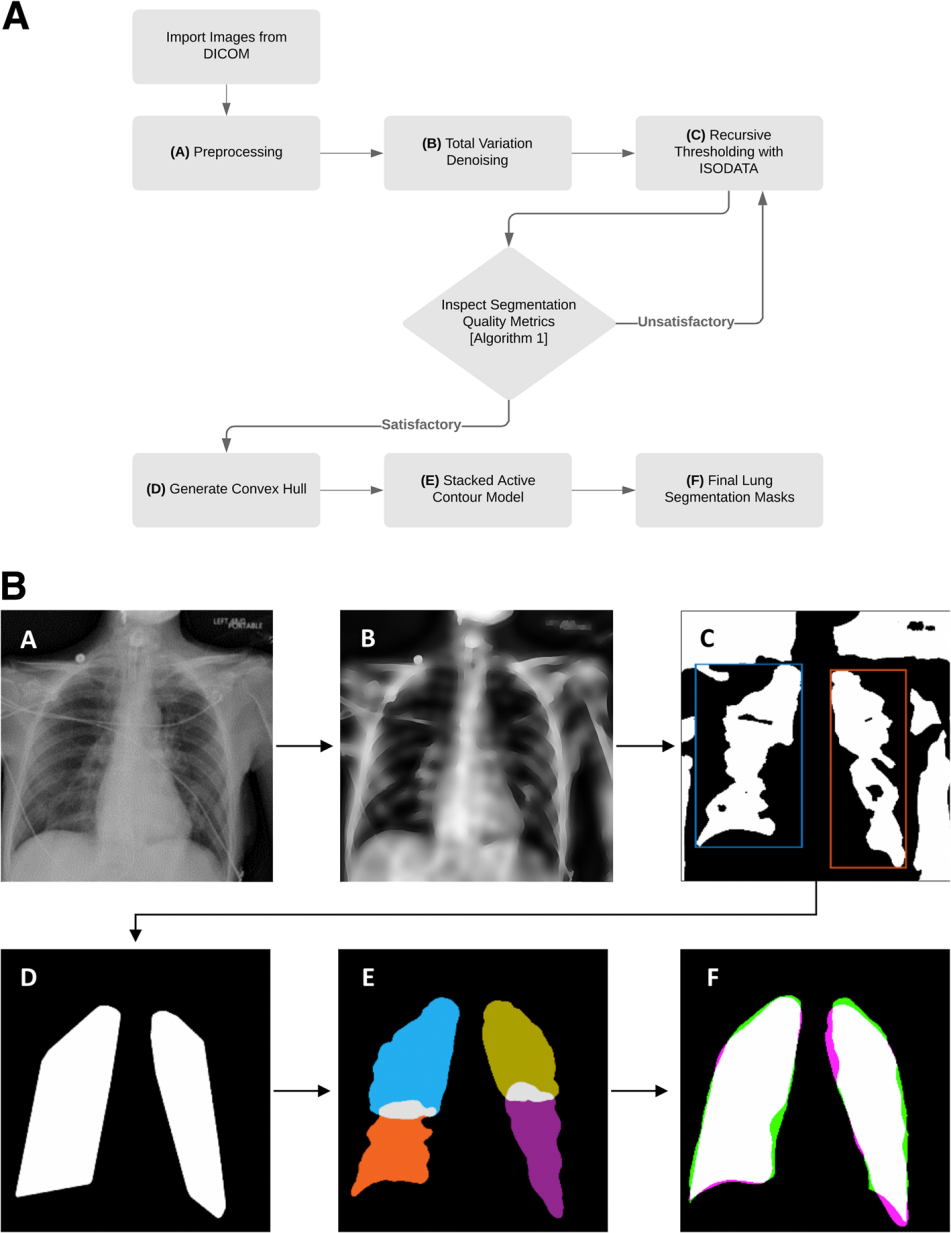
General outline of the proposed total-variation based active contour (TVAC) method. (**a**) An example source image containing a few wires from a patient diagnosed with acute hypoxic respiratory failure is shown. This image is normalized with contrast-limited adaptive histogram equalization (CLAHE) at this step. (**b**) Total variation denoising is used to diffuse wires while preserving edges of the lungs. (**c**) The denoised image is binarized with recursive thresholding and initial lung segments are extracted. (**d**) Convex hulls are generated from the extracted lung regions to enclose the lung fields and capture regions lost during binarization. (**e**) Lungs are partitioned into quadrants; each is individually processed with the stacked active contour model to better capture “difficult” regions such as the apex and costophrenic recess. (**f**) Final output of the lung segmentation algorithm. Green represents the ground truth, magenta shows the algorithm’s segmentation output, and white illustrates overlap of the two – indicating regions that are correctly segmented. This example has a Dice coefficient of 0.9407

ROF denoising is used to remove irrelevant details and artifacts (e.g. electrocardiographic leads and prosthetic devices) from chest radiographs. Unlike blurring, total variation denoising preserves sharp edges such as the boundary of the lungs. The processed image retains most of the structurally large, well-defined regions of the original image while removing unwanted objects of fine scale and discontinuous variations. This workflow is illustrated in Fig. 1a and b.

#### Binarization with recursive thresholding and lung field identification

After denoising, the lung fields are localized through binarization of the image with a recursive threshold. Binarization assumes that an image contains two classes of pixels following a bi-modal distribution, where the foreground (region of interest) and background pixels can be distinguished by finding an optimal threshold separating the two classes. To determine this optimal threshold for global binarization, *θ*_*k*_, the Iterative Self-Organizing Data Analysis Technique (ISODATA) [23] is used.

First, the histogram is initially segmented into two parts using a starting threshold (*θ*_0_) at half the maximum dynamic range. The mean of the values associated with the foreground pixels $\left (\mu _{f,\theta _{0}}\right)$ and background pixels $\left (\mu _{b,\theta _{0}}\right)$ is calculated. An updated threshold value *θ*_1_ is calculated as the average of these two sample means. This method is repeated until the updated threshold value doesn’t change anymore. This process is formalized as:

3 $$ \theta_{k} = \frac{\mu_{f,\theta_{k}-1} + \mu_{b,\theta_{k}-1}}{2} \ \text{until} \ \theta_{k}=\theta_{k-1}  $$

The denoised image is then binarized with threshold *θ*_*k*_.

After binarization, morphological area opening is performed to remove small objects corresponding to artifacts from binarization. To extract the left lung, an object whose centroid is nearest to the upper right half of the image is selected; to extract the right lung, another object whose centroid is nearest to the upper left half of the image is selected. The binary masks (regions containing the object of interest) extracted from this process are shown in Fig. 1c. Both objects are assessed for quality of segmentation and similarity comparison, summarized in Algorithm 1, to ensure that they accurately correspond to the two lung fields. The parameters utilized in Algorithm 1 can be varied as needed to implement this method for similar applications. The specific values used for this experiment are provided in Table 3. These values generated reasonable results and demonstrated robustness to variations in analysis. In particular, we increased and decreased these values by 10% and observed that these changes did not have a significant impact on the results. If the masks violate more than 1 of these criteria, threshold *θ*_*k*_ is reduced by 5% and binarization is repeated. This process of recursively reducing threshold *θ*_*k*_ is repeated until all but one quality criteria are satisfied.

**Table 3:** Parameters for Algorithm 1

***α***	**0.98**
*β*	135
*γ*	1/3
*δ*	1/100

Although a wide range of parameters were tested, these are the specific values used in this experiment. These values generated reasonable results and demonstrated robustness to variation in analysis - even when the values were increased or decreased by 10%.

Convex hulls are then generated from both masks to enclose the lung fields. This geometric representation of the lung fields, shown in Fig. 1d, is the smallest convex polygon shaped by vertices of the previous mask and is designed to capture interior regions that weren’t included during binarization.


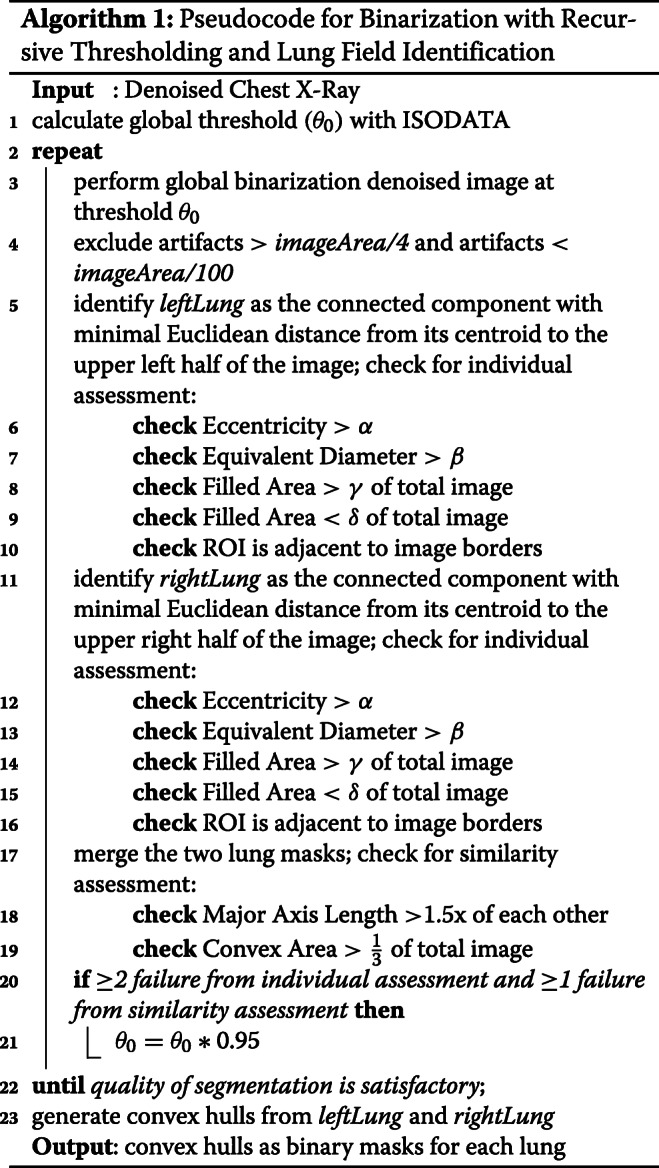


#### Stacked active contour model

Following denoising and lung field segmentation the two convex hulls are then further refined to better capture the shape of the lungs. A standard active contour model (ACM) [24] is able to use these templates as a deformable spline, allowing the convex hulls to “stretch” and better fit to the pleural lining of the lung. However, we found that using the lung field as the template yielded unfavorable results and incomplete segmentation, particularly with respect to the costophrenic recess and in peripheral regions. To overcome this obstacle, we developed a stacked active contour model where the lung quadrants, rather than the whole lung field, are used as templates to better capture these peripheral regions. Standard ACM uses a pre-defined number of consecutive iterations to expand or contract based on minimization of energy and other constraint forces. The proposed ACM model sequentially “stacks” 50 iterations of parameterized contour expansions, followed by 50 iterations of parameterized contractions. This process is repeated 20 times, resulting in a total of 1000 iterations.

**Fig. 2:**
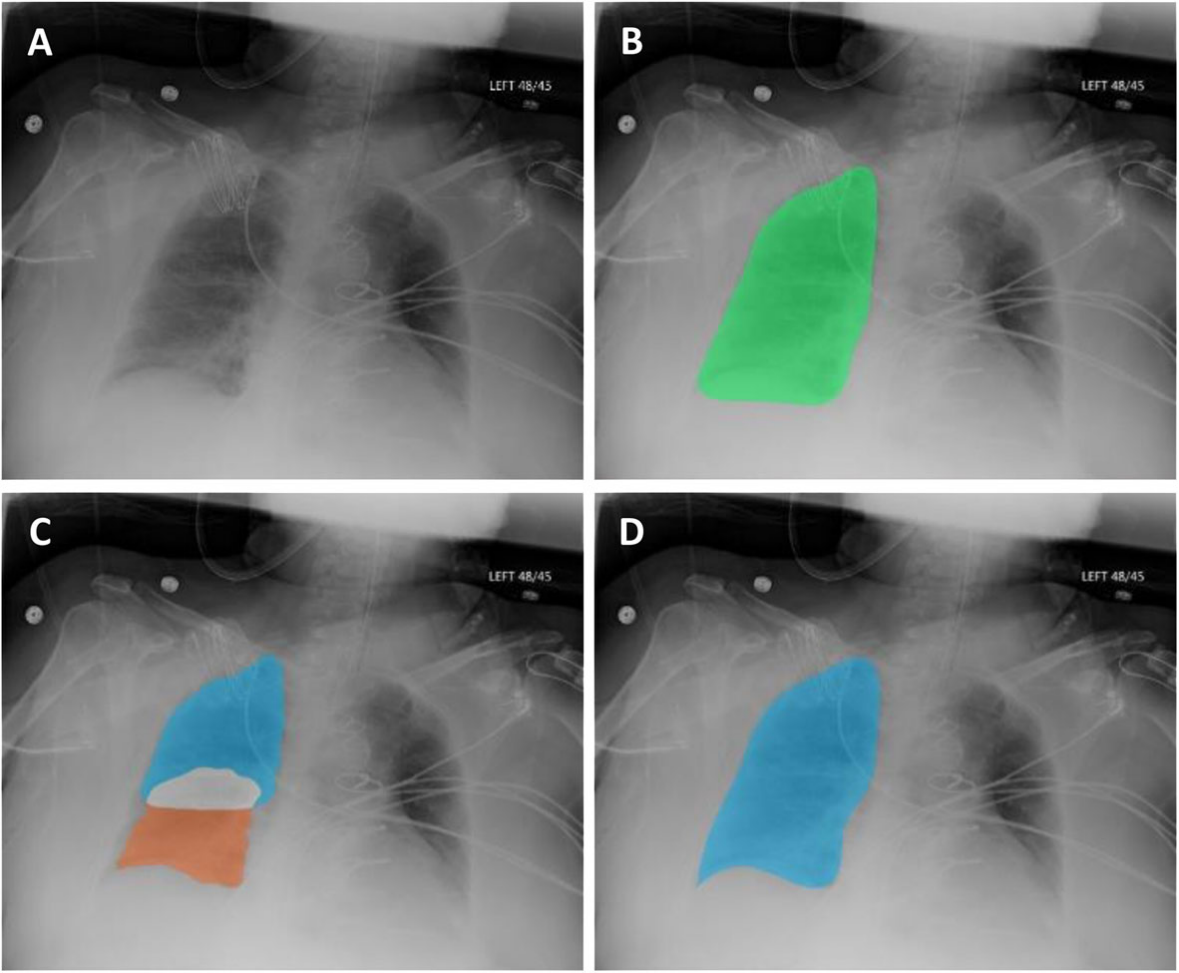
Segmentation with the stacked active contour model. (**a**) An example source image is shown for reference. (**b**) When the final segmentation mask is processed with a standard active contour model, areas of incorrect segmentation can be systematically observed – most commonly, at the right lung’s costophrenic recess and regions adjacent to the diaphragm. (**c**) Quadrant-based processing with a stacked active contour model shows better deformation and contouring to peripheral boundaries. (**d**) Final output for segmentation of the right lung after combining the upper and lower quadrants and applying a smoothing filter

The two masks from the upper and lower quadrants of each side are then combined to reconstruct the lung fields. This final step is shown in Fig. 2c and d for reconstruction of the right lung field. A smoothing filter is applied to remove jagged edges on the mask boundary.

### Lung segmentation from chest x-rays obtained from critically ill patients

### Comparison with state-of-the-art lung segmentation algorithms

A deep learning approaching using U-Net, a convolutional neural network (CNN) architecture designed for biomedical image segmentation [12], was included as a “state-of-the-art” benchmark. A widely-used algorithm based on random walks [13] and another established shape-based “active spline” model [14] were also included for comparison with conventional image processing methods. These algorithms were selected on the basis of having excellent performance results on publicly available databases, being widely cited, and having an available codebase.

The U-Net CNN was implemented with Keras [25] using the TensorFlow backend and trained on both the JSRT and Montgomery datasets with 5-fold cross-validation. To further extend this analysis, additional experiments were conducted with the U-Net trained on JSRT, Montgomery, and 50% of Michigan Medicine data (including adult ARDS, adult severe ARDS, and pediatric ARDS) to “fine-tune” the model so that it encounters an even greater variety in patient population and heterogeneity of disease in the target dataset.

We used a modified implementation of the random walker algorithm designed for unsupervised lung segmentation. This version relies on extracting horizontal intensity profiles to intuitively match a pre-designed template to identify anatomical regions of the x-ray and accordingly place seed points for segmentation. The active spline model used in this study is a combined point distribution model and centripetal-parameterized Catmull-Rom spline for lung segmentation. This “template matching” method uses a fixed set of points resembling a generalized shape of the lungs and adapts this template to capture the lung fields from chest x-rays. Additional details for these methods are published in previous works [12–14].

### Evaluation metric

We used the Sørensen–Dice coefficient, a statistical validation method based on spatial overlap to measure the degree of similarity between the algorithm’s segmentation and ground truth reference as annotated by multiple clinicians [26, 27]. Given two sets X and Y representing the segmentation output and ground truth, respectively, the Dice coefficient is defined as:

4 $$ Dice(A,B) = \frac{2TP}{2TP+FP+FN}  $$

For this study, a Dice coefficient under 0.70 is recognized as failed lung segmentation. This value is determined, through our experience from similar studies, as the lowest acceptable level of segmentation correctness for effective feature extraction and sufficient for machine learning.

### Violin plot

In addition to reporting summary statistics, we also present our experimental results with a violin plot generated by a kernel density estimate of all the results [28]. These plots are essentially mirrored density plots and enables a comparison of algorithm performance, in terms of Dice coefficient, across patient populations. The thicker part of a violin plot indicates higher frequency, and the thinner part implies lower frequency. Violin plots with “longer tails” represent algorithms that more often failed to accurately segment a patient’s lungs within a population.

## Results

**Fig. 3:**
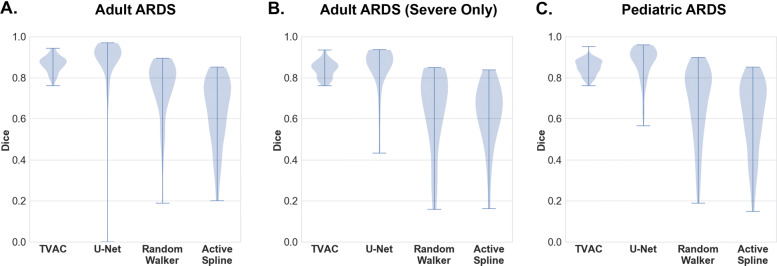
Violin plot of segmentation results from the Michigan Medicine dataset for (**a**) the adult ARDS data, (**b**) the adult ARDS dataset comprising of only severe cases, and (**c**) pediatric ARDS dataset

Summary statistics of lung segmentation performance (mean, min, and standard deviation of Dice coefficient) on the entire Michigan Medicine dataset, stratified by different patient cohorts, from all four algorithms are reported in Table 4. The results in Table 5 provide summary statistics from 50% of the Michigan Medicine (held-out) dataset, when the other 50% is used for “fine-tuning” the U-Net algorithm. Violin plots are also provided in Fig. 3 to better visualize the distribution and density of the reported results. On all critically ill patient cohorts, TVAC and U-Net outperformed the random walker and active spline model. Although the TVAC model and U-Net show comparable mean Dice coefficients, the TVAC algorithm maintained more consistency in standard deviation and reliable performance (higher lowest Dice coefficient) across all 3 patient groups.

**Table 4:** Lung Segmentation Accuracy for the Michigan Medicine Dataset

	**Adult (n = 100)**			**Adult Severe ARDS (n = 25)**			**Pediatric (n = 100)**		
	**Dice (mean)**	**Dice (min)**	**Standard Deviation**	**Dice (mean)**	**Dice (min)**	**Standard Deviation**	**Dice (mean)**	**Dice (min)**	**Standard Deviation**
**TVAC**	0.8661	0.7616	0.0392	0.8416	0.7614	0.0409	0.8507	0.7526	0.0375
**U-Net**	0.8885	0.0001	0.1177	0.8482	0.4332	0.1181	0.8732	0.5645	0.0853
**Random Walker**	0.7446	0.1497	0.1882	0.6463	0.1596	0.1619	0.6731	0.1892	0.1778
**Active Spline**	0.6401	0.2003	0.1670	0.6229	0.1583	0.1583	0.6137	0.1491	0.1760

Data are mean with minimum and standard deviation reported for each algorithm on different patient populations. TVAC = Total Variation-based Active Contour, Dice = Sørensen–Dice coefficient, ARDS = acute respiratory distress syndrome

**Table 5:** Lung Segmentation Accuracy for the Michigan Medicine Dataset (50% Held-Out) with U-Net Fine Tuning

	**Adult (n = 50)**			**Adult Severe ARDS (n = 13)**			**Pediatric (n = 50)**		
	**Dice (mean)**	**Dice (min)**	**Standard Deviation**	**Dice (mean)**	**Dice (min)**	**Standard Deviation**	**Dice (mean)**	**Dice (min)**	**Standard Deviation**
**TVAC**	0.8608	0.7790	0.0439	0.8301	0.7614	0.0423	0.8467	0.7827	0.0364
**U-Net**	0.8953	0.4842	0.0975	0.8188	0.0001	0.2685	0.8829	0.4277	0.1403
**Random Walker**	0.7422	0.3898	0.1238	0.6711	0.3405	0.1448	0.6681	0.2692	0.1675
**Active Spline**	0.6475	0.2003	0.1743	0.6431	0.3410	0.1270	0.6203	0.2003	0.1814

Data are mean with minimum and standard deviation reported for each algorithm on different patient populations. TVAC = Total Variation-based Active Contour, Dice = Sørensen–Dice coefficient, ARDS = acute respiratory distress syndrome

### Experimental results on critical care patients (Michigan Medicine dataset)

The TVAC algorithm was able to successfully segment lungs from chest radiographs of all critically ill patient cohorts; the lowest Dice coefficient reported was 0.75 from the pediatric cohort. Without fine-tuning, the U-Net has a total of 12 lung segmentation failures from the entire Michigan Medicine test set: the algorithm was unable to segment 4% of the adult cohort, 8% of the adult severe ARDS cohort, and 6% of the pediatric cohort. With fine-tuning and exposure to a subset of Michigan Medicine’s data in its training set, the U-Net has a total of 12 lung segmentation failures from the 50% held-out test set: the algorithm was unable to segment 8% of the adult cohort, 15% of the adult severe ARDS cohort, and 12% of the pediatric cohort.

In comparison, the random walker algorithm was observed to have 83 unsuccessful lung segmentations, failing 26% of the adult cohort, 44% of the adult severe ARDS cohort, and 46% of the pediatric cohort. The most failures were observed from the active spline model, which reported a total of 130 failures from 55% of the adult cohort, 56% of the adult severe ARDS cohort, and 58% of the pediatric cohort.

### Lung segmentation from chest x-rays obtained from critically ill patients

**Fig. 4:**
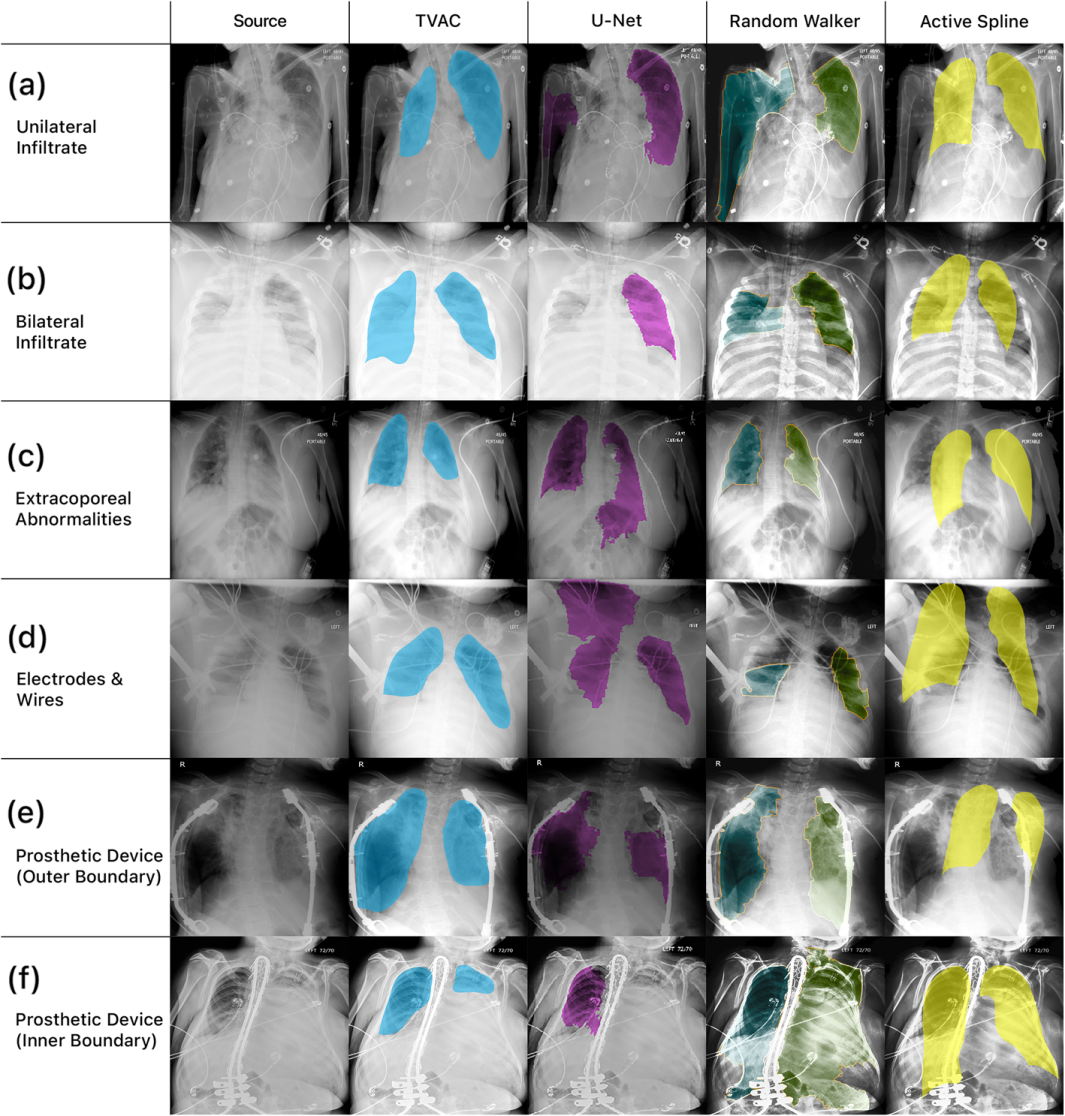
Lung segmentation from chest radiographs of hospitalized patients at Michigan Medicine. This figure illustrates the qualitative difference among algorithms and focuses on how they fail in different clinical scenarios, including (**a**) manifestations of unilateral infiltrate (**b**) bilateral lung opacities (**c**) extracorporeal abnormality from an unrelated comorbidity in the abdomen (**d**) electrocardiographic leads overlying the lung fields (**e**) a prosthetic device obscuring the outer boundary of the lungs and (**f**) a prosthetic device interfering with the inner boundary of the lungs

In Fig. 4, all four algorithms and their final lung segmentation from chest x-rays of critically ill patients in the Michigan Medicine dataset are shown. These examples were selected to present common pathological findings and characteristics of more complex chest x-rays from hospitalized patients. These visually qualitative results are presented to provide insight into the difficulty of this task and why these algorithms may fail.

Nearly all segmentation methods performed well on lung fields that were clearly defined, unobscured by medical equipment, and absent or with minimal manifestation of any pathological conditions. In the presence of abnormalities, such as pulmonary infiltrate in Fig. 4a or lung opacities in Fig. 4b, U-Net and the random walker algorithm both failed to produce acceptable results on these examples. Patients suffering from traumatic injury may also present with multiple abnormalities from these comorbidities. An example of extracorporeal abnormality in the abdomen is shown in Fig. 4c. These types of issues may be problematic for deep learning approaches, which are rigorously trained to identify a specific pattern representation and may struggle when present with an unexpected example outside of what the algorithm has been trained on.

The presence of medical equipment present throughout the chest x-ray is also problematic. Figure 4d shows an example with electrocardiographic leads and wires, which have visual characteristics comparable to the lung field boundaries (e.g. edges that are well-defined, bright, and elongated). In this example, U-Net recognizes the wires as an extension of the lung boundary and overextends the final segmentation mask of the right lung field into the patient’s shoulder region. The random walker algorithm identifies the wire as lung boundary and produces two lung segmentation masks truncated at where the wires overlay the lung fields. Additional examples of obscuring medical equipment are shown in Fig. 4e and f, we observe that both the random walker algorithm and active spline model fails for similar reasons as previously mentioned.

### Experimental results on JSRT and Montgomery datasets

Summary statistics of lung segmentation performance on both the JSRT and Montgomery datasets from our proposed algorithm (TVAC), the U-Net CNN, Random Walker, and Active Spline Model are reported in Table 6. On these two datasets containing standardized chest radiographs from previous studies, all four algorithms perform relatively well. The U-Net CNN reports the best performance (Dice: 0.98 ±0.01 for JSRT, 0.97 ±0.03 for Montgomery) of lung segmentation from these two datasets, followed by our proposed TVAC method (Dice: 0.95 ±0.03 for JSRT, 0.96 ±0.03 for Montgomery), the Random Walker algorithm (Dice: 0.88 ±0.06 for JSRT, 0.88 ±0.07 for Montgomery), and the Active Spline Model (Dice: 0.88 ±0.08 for JSRT, 0.87 ±0.08 for JSRT).

**Table 6:** Lung Segmentation Accuracy for the JSRT and Montgomery Dataset

	**Adult (n = 247)**			**Montgomery (n = 138)**		
	**Dice (mean)**	**Dice (min)**	**Standard Deviation**	**Dice (mean)**	**Dice (min)**	**Standard Deviation**
**TVAC**	0.9501	0.8488	0.0297	0.9569	0.8566	0.0251
**U-Net**	0.9817	0.9500	0.0012	0.9694	0.8442	0.0267
**Random Walker**	0.8809	0.4973	0.0576	0.8783	0.5084	0.0729
**Active Spline**	0.8790	0.0001	0.0783	0.8672	0.3835	0.0826

Data are mean with minimum and standard deviation reported for each algorithm on different patient populations. TVAC = Total Variation-based Active Contour, Dice = Sørensen–Dice coefficient, ARDS = acute respiratory distress syndrome

## Discussion

In this study, we developed a lung segmentation algorithm that would perform well on both publicly available datasets from retrospective research studies and on real-world data obtained from hospital and inpatient care, especially from critically ill patients. We demonstrate that our TVAC algorithm is capable of accurate and reliable lung segmentation from chest x-rays in the Michigan Medicine dataset comprising of hospitalized patients, of varying demographics and age groups, diagnosed with moderate hypoxia, acute hypoxic respiratory failure, or ARDS. Furthermore, we also evaluated TVAC on publicly available chest x-rays from the JSRT and Montgomery datasets to benchmark our proposed method with multiple state-of-the-art lung segmentation algorithms.

Many published algorithms and software platforms capable of lung segmentation exist [2–8]. However, nearly all of them have only been evaluated on chest radiographs where the lungs exhibit minimal or no pathological conditions [29]. Segmentation of normal, healthy lungs can be fairly straightforward, as the black pixels of the lung fields can be readily delineated from the white pixels of peripheral anatomic regions [30]. This task becomes challenging when segmenting lungs from chest x-rays of critically ill patients diagnosed with a lung disease or severe condition, such as ARDS, pneumonia, and pulmonary edema. These injuries tend to manifest with a white diffuse appearance [31–33] that may be incorrectly recognized by many algorithms as regions outside the lungs, as these attenuating characteristics are similar to the soft tissue of nearby anatomic structures. As a result, consolidation along the pleural margin of the lungs may generate an erroneous delineation and incorrect segmentation. These complications are also present in related applications and orthogonal studies (e.g. detection of consolidation) involving complex chest x-rays from hospitalized patients [11].

Furthermore, medical equipment such as wires, tubes, pacemakers, and various prosthetic devices can obscure lung fields on chest x-rays [9]. These objects are characteristic of CXRs obtained from hospitalized patients or during inpatient care, which may contain a diverse array of medical equipment used to monitor and treat patients [34]. These items appear as connected regions of high pixel intensity with strong edges, often interfering with edge detection of the lung’s pleural space and resulting inaccurate boundaries. Because these objects don’t typically appear in CXRs obtained from outpatient care or controlled studies, it is therefore essential to include these types of complex data from clinical and hospital settings in the evaluation set of any automatic lung segmentation algorithm. These physiological abnormalities and noise from medical devices can hinder segmentation methods using lung models that have been computed on healthy lungs only [35]. Because of this, we also sought to investigate the efficacy and reliability of these algorithms on our Michigan Medicine dataset.

Despite the high overall performance of the deep learning approach, our experimental results demonstrate that U-Net can be inconsistent and suffers from numerous lung segmentation failures. Based on the violin plots as well as results of segmentation on the JSRT and Montgomery datasets, we can infer that U-Net performs very well on the types of x-rays it has encountered before. However, when new, unseen examples of disease and noise are shown, the CNN is unable to generalize pattern recognition for these challenging lung fields. Even when the U-Net is fine-tuned with 50% of all available Michigan Medicine so that it encounters an even greater variety in patient population and heterogeneity of disease in the target dataset, the same segmentation issues still persist - namely, failure to recognize the lung boundaries due to interference from medical equipment or gross abnormalities present on the image. These results suggest that although the U-Net is very capable of excellent segmentation, robustness of the deep learning approach needs to be improved before it is practical for clinical use.

The “template matching” active spline model suffers from similar generalizability issues as U-Net. On chest x-rays with well-defined lung boundaries, the algorithm is capable of producing excellent masks. However, when lung fields and pleural regions are obscured by injury (e.g. collapsed lung), the template matching attempt usually fails [29, 36]. When developing TVAC, we also take into consideration the issues of deployability, usability, and trustworthiness from the perspective of a healthcare provider. Missing a few pixels is better than missing an entire lung field – especially if the algorithm is extended and applied to subsequent clinical tasks (e.g. using lung segmentation as a preprocessing step for prediction of acute respiratory distress syndrome, pneumonia, or sepsis). Making a clinical decision based on inaccurate information could be extremely dangerous for the patient’s outcome and we believe that healthcare providers would likely opt for a more consistent system in lieu of one with a slightly higher mean performance benchmark but less reliability.

We recognize that there are several limitations to this study. The cohort sample sizes were relatively small, which limited the extent of stratified analysis, such as looking at challenges in segmentation grouped by type of lung injury or in the presence of a specific treatment/medical device. Furthermore, due to the limited amount of data available from Michigan Medicine, we were not able to train the U-Net on this dataset. Therefore, the U-Net was trained on both the JSRT and Montgomery datasets combined (evaluated with 5-fold cross validation) and we thus relied on transfer learning for generalization of this CNN to the Michigan Medicine dataset. The use of significantly larger training databases of CXR with heterogeneous characteristics in future studies may improve the performance of the U-Net CNN. Another limitation to note is that ground truth annotations of the lung fields were provided by critical care physicians instead of radiologists. Although we don’t believe this has affected our study, we do acknowledge that many similar studies involving ground truth from radiographs typically relies on a radiologist, or the supervision of a radiologist, to correctly annotate the image.

## Conclusions

This study introduced a lung segmentation method capable of robust and reliable performance on multiple patient populations, including critically ill adult and pediatric cohorts. By evaluating TVAC and other state-of-the-art approaches on the Michigan Medicine dataset, we hope to highlight the advantages and shortcomings of each approach as well as understand the challenges of lung segmentation within a true inpatient population. Although the proposed TVAC model and U-Net show comparable mean Dice coefficients, the TVAC algorithm maintained more consistency in standard deviation and reliable performance across all 3 patient groups. We also observe that the other standard lung segmentation algorithms can be inconsistent and suffer from numerous lung segmentation failures, especially in the presence of notable pathological findings and noise from medical equipment. From a clinical perspective, it is especially important that these clinical decision support systems are highly reliable to ensure healthcare providers that the algorithm will consistently perform as expected, even in the most rigorous tasks.

We believe this paper makes a significant contribution towards evaluation of lung segmentation approaches on real-world, complex chest x-rays and hope our contribution will supplement the role of clinical decision support systems by guiding the development of automated methods for pulmonary analysis. In the future, we plan to conduct a larger evaluation and include more respiratory conditions (e.g. pneumonia and chronic obstructive pulmonary disease) by obtaining additional data from Michigan Medicine and other hospital systems. We also plan to implement TVAC with feature extraction techniques and machine learning algorithms for further downstream analysis and detection of these aforementioned lung diseases.

## Data Availability

The data used in this study could be made available under a data sharing agreement with the University of Michigan.

## Abbreviations

ARDS: Acute respiratory distress syndrome
CXR: Chest x-ray
TVAC: Total variation-based active contour
JSRT: Japanese society of radiological technology
PaO_2_: Partial pressure of oxygen
FiO_2_: Fraction of inspired oxygen
CLAHE: Contrast-limited adaptive histogram equalization
ROF: Rudin, Osher, and Fatemi
PDHG: Primal-dual Hybrid gradient
ISODATA: Iterative self-organizing data analysis technique
ACM: Active contour model
CNN: Convolutional neural network
